# Early Surgical Resection in Pediatric Patients with Localized Ileo-Cecal Crohn’s Disease: Results of a Retrospective Multicenter Study

**DOI:** 10.3390/jcm14020404

**Published:** 2025-01-10

**Authors:** Isabella Madaffari, Edoardo Maria Muttillo, Alice La Franca, Fanny Massimi, Giorgio Castagnola, Alessandro Coppola, Silvia Furio, Marisa Piccirillo, Alessandro Ferretti, Maurizio Mennini, Pasquale Parisi, Denis A. Cozzi, Silvia Ceccanti, Enrico Felici, Pini Prato Alessio, Gabriele Lisi, Maria Teresa Illiceto, Isabella Sperduti, Giovanni Di Nardo, Paolo Mercantini

**Affiliations:** 1 Department of Medical Surgical Science and Translational Medicine, Sant’ Andrea University Hospital, Sapienza University of Rome, 00185 Roma, Italy; edoardomaria.muttillo@uniroma1.it (E.M.M.); alicelafranca@gmail.com (A.L.F.); fanny.massimi@uniroma1.it (F.M.); giorgiocastagnola@hotmail.com (G.C.); paolo.mercantini@uniroma1.it (P.M.); 2 Department of General Surgery, Sapienza University of Rome, 00185 Roma, Italy; coppola.chirurgia@gmail.com; 3 NESMOS Department, Sant’Andrea University Hospital, Sapienza University of Rome, 00185 Roma, Italy; sivifurio@gmail.com (S.F.); marisa.piccirillo@outlook.it (M.P.); alessandro.ferretti@uniroma1.it (A.F.); maurizio.mennini@gmail.com (M.M.); pasquale.parisi@uniroma1.it (P.P.); giovanni.dinardo@uniroma1.it (G.D.N.); 4 Pediatric Surgery Unit, Sapienza University of Rome, AOU Policlinico Umberto I, 00185 Roma, Italy; da.cozzi@uniroma1.it (D.A.C.); silvia.ceccanti@uniroma.it (S.C.); 5 Pediatric and Pediatric Emergency Unit, Children Hospital, AO SS Antonio e Biagio e Cesare Arrigo, 15121 Alessandria, Italy; enrico.felici@ospedale.al.it; 6 Pediatric Surgery Unit, Children Hospital, AO SS Antonio e Biagio e Cesare Arrigo, 15121 Alessandria, Italy; apini@ospedale.al.it; 7 Department of Medicine and Aging Science, “G. d’Annunzio” University of Chieti-Pescara, 2 Pediatric Surgery Unit, “Santo Spirito” Hospital of Pescara, 66100 Pescara, Italy; gabriele.lisi@unich.it; 8 Pediatric Gastroenterology and Digestive Endoscopic Unit, Department of Pediatrics, “Santo Spirito” Hospital of Pescara, 65124 Pescara, Italy; mtilli600@yahoo.it; 9 Biostatistical Unit, Clinical Trials Center, IRCSS Regina Elena National Cancer Institute, 00144 Rome, Italy; isabella.sperduti@ifo.it

**Keywords:** Crohn’s disease, surgery, laparoscopy, inflammatory bowel disease

## Abstract

**Background**: Crohn’s disease (CD) is an inflammatory bowel disease (IBD) that also affects pediatric patients. It frequently presents as a localized disease, affecting the ileocecal area, ileum, or colon. It requires targeted therapy to achieve a good quality of life and long-term control of disease activity. Despite multiple medical therapies available, several patients benefit from surgical treatment. The aim of our study is to demonstrate how an early surgical approach can bring an improvement in disease activity, evaluating the Simple Endoscopic Score for Crohn’s Disease (SES-CD) and the Pediatric Crohn’s Disease Activity Index (PCDAI). **Methods**: A retrospective multicenter study was carried out from 2008 to 2023, including 29 patients, affected by localized CD. These data were analyzed: demographics, SES-CD, and PCDAI, before and after surgery. The differences between groups were analyzed using Student’s t-test for continuous variables, and Pearson’s Chi-squared test or Fisher’s exact test for categorical variables. **Results**: The SES-CD significantly decreased from 12 (median, range 1–15) to 0 (median, range 0–6) (*p* < 0.0001) and the PCDAI decreased from 30 (median, range 10–50) to 0 (median, range 0–15) (*p* < 0.0001). The rate of patients receiving enteral nutrition decreased from 51.7% preoperatively to 0% postoperatively (*p* = 0.0001). The rate of antibiotic use decreased from 13.8% to 0% (*p* = 0.0001). The rate of patients receiving ≥2 drugs decreased from 10.3% to 0% (*p* = 0.0001). **Conclusions**: The early surgical approach can be considered an excellent therapeutic strategy in patients with localized CD. Both parameters examined, SES-CD and PCDAI, demonstrated a clear improvement in the endoscopic images and in disease activity.

## 1. Introduction

CD is a chronic transmural IBD that usually affects the distal ileum and colon, but it may occur in any part of the gastrointestinal tract. Symptoms include diarrhea and abdominal pain but also abscesses, internal and external fistulas, and bowel obstruction [1]. The incidence of CD in children is increasing, with values ranging from 2.5 to 11.4 per 100,000 and a prevalence of 58 per 100,000. Approximately 20 to 25% of patients who present with IBD are children younger than 18 years, and 80% are in adolescence [2]. Diagnosis of IBD should be based on medical history, physical and laboratory examination, esophagogastroduodenoscopy (EGD), ileocolonoscopy with histology and imaging of the small intestine (magnetic resonance enterography, wireless capsule endoscopy, ultrasonography). A peculiar feature of CD is ileitis in the presence of a normal-looking cecum, or the presence of ileal fissure ulcers [3]. In about a third of cases, the disease occurs in the ileocecal area, while in the remaining cases, it involves the ileum or colon [4]. We speak of localized disease when it occurs with an extension <30 cm. [5].

CD, being a chronic and progressive course, requires early intervention and intensive monitoring to prevent complications in these patients [6]. The main treatment goals in pediatric patients are as follows: reduce symptoms, optimize growth, and improve quality of life while minimizing drug toxicity [7].

The usual approach is “step-up” from 5-aminosalicylates (5-ASA) to corticosteroids (CS) and from immunomodulators (IM) to biologics. Nevertheless, in patients with severe disease, a “top-down” approach is more effective [8]. According to the Paediatric IBD Porto Group of ESPGHAN, surgery may be considered in the therapeutic procedure, when the active disease is limited to short intestinal segments despite optimized medical treatment and also in children who have a reduced growth velocity for bone age despite optimized medical and nutritional therapy [9]. For the European Crohn’s and Colitis Organization (ECCO), indications for surgical treatment are fibrotic stenosis (which cannot be treated endoscopically), penetrating disease with symptomatic internal or enterocutaneous fistula, disease refractory to medical therapy or which presents a failure to respond to biological drugs, and finally, the development of neoplasia [10,11]. Similarly, the American College of Gastroenterology (ACG) guidelines recommend surgery only in patients with complications such as obstruction, intestinal perforation, and abscess formation, or in case of disease refractory to therapy [12]. Meanwhile, The British National Institute for Health and Care Excellence (NICE) guidelines recommend surgery at an early stage of disease in patients with distal ileum involvement, with or without impaired growth in children and refractory disease [13].

Wherever possible, laparoscopy should be the preferred approach. It results in reduced morbidity, shorter hospitalization, reductions in adhesions, and hernia formation [14]. Extended small bowel resections should be avoided as they can lead to the development of short bowel syndrome, even if, when a pancolic disease is present, the most appropriate treatment choice is a subtotal colectomy [9]. Occasionally, CD can present with acute complications requiring emergency surgery in approximately 6–16% of cases. [11,15].

In the Laparoscopic Ileocolic Resection Versus Infliximab Treatment of Recurrent Distal Ileitis in Crohn’s Disease (LIR!C) randomized clinical trial, the improvement in quality of life after ileocolic resection (ICR) was similar to that achieved with Infliximab [16].

The aim of our study is to evaluate if an early surgical approach can bring a substantial improvement in disease activity and, thus, in patients’ quality of life. The primary endpoint is to evaluate the SES-CD and PCDAI before and after surgery, while as a secondary endpoint, we evaluated therapeutic changes in these two periods, such as enteral nutrition, the use of antibiotics, and the use of combined therapies with more than two drugs, as well as the use of anti-inflammatories, azathioprine, and biological drugs.

## 2. Materials and Methods

We performed a retrospective observational study selecting pediatric patients with CD who underwent surgery at Sant’ Andrea Hospital (Rome, Italy), Policlinico Umberto I (Rome, Italy), Ospedale Infantile “Cesare Arrigo” (Alessandria, Italy), and Ospedale Civile dello Spirito Santo (Pescara, Italy) from 2008 to 2023. The inclusion criteria were patients with localized CD, reported from pathological examination, treated with surgery, and aged ≤18 years. The indication for surgery was the presence of at least one of the following: stenosis, fistula, and severe disease unresponsive to medical treatment. On this point, clinical response was defined as PCDAI ≤10 [17]. The exclusion criteria were patients with age >18 years. Finally, 29 patients were selected for the analysis. Before surgery, 24 patients were evaluated through Magnetic Resonance Imaging (MRI) and 5 through a Computed Tomography (CT) scan. Two scores were used, both before and after surgery: a clinical one, the PCDAI, and an endoscopic one, the SES-CD, to detect endoscopic disease activity.

The SES-CD is based on the evaluation of five defined bowel segments (rectum, sigma + descending colon, transverse colon, ascending colon, and terminal ileum), and in these segments, the presence and size of ulcerations and the extent of the inflammatory area and stenosis were assessed, then classified in severity as a score of 0–3. The scores for each individual segment are added together as a sum score (Table 1) [18]. The PCDAI includes history items (abdominal pain and number of liquid stools), general wellbeing, physical examination items (abdominal examination, perirectal disease, extra-intestinal manifestations, weight, and height at diagnosis), and three laboratory tests (hematocrit, albumin, and erythrocyte sedimentation rate). These items are scored on a three-point scale (0, 5, or 10 points) except for hematocrit and erythrocyte sedimentation rate, which are scored as zero, 2.5, or 5 points. PCDAI scores can range from zero to 100, with higher scores indicating a disease that is more active (Table 2) [19,20]. Endoscopic score (SES-CD) was classified in remission (<2), mild (3–6), moderate (7–14), and severe (≥15); while the clinical one (PCDAI) was classified in remission (<10), mild (10–27), moderate (28–37), and severe (≥38).

### Statistical Methods

Descriptive statistics were used to summarize the characteristics of the study population, including intraoperative and perioperative data.

Continuous variables were described as median and range or mean and SD, while categorical variables as units and percentages. The differences between groups (before and after surgery) were analyzed using Pearson’s Chi-squared test or Fisher’s exact test for categorical variables and Student’s *t*-test for continuous variables. A *p*-value < 0.05 was considered statistically significant. All statistical analyses were performed using R (version 4.3.1; R Foundation for Statistical Computing, Vienna, Austria).

## 3. Results

In the examined population of 29 patients, 19 were males (65.5%) and 10 were females (34.5%).

The median age at diagnosis was 12 years (range 5–16). No comorbidities were detected in the sample examined. Medical treatment was quite heterogeneous: Adalimumab (ADA) in 8 patients (27%), ADA + Exclusive Enteral Nutrition (EEN) in 6 (20%), EEN alone in 3 (10%), Azathioprine (AZA) in 3 (10%), EEN + Antibiotic in 3 (10%), AZA + Infliximab in 3 (10%), EEN + Budesonide in 1 (3.4%), EEN + AZA in 1 (3.4%), and Infliximab alone in 1 (3.4%). Patients have been under medical treatment for a median of 10 months (range 1–144) before surgery. Considering the preoperative inflammation parameters, 19.23% of patients had neutrophilic leukocytosis, and 96.55% had increased CRP (mean 16.83 mg/dL; SD 22.08 mg/dL). At the time of intervention, the median age was 14 years (range 8–20), and the patients included in the study had localized disease, which means <30 cm of intestinal tract involvement [5]. In detail, in our cohort, in 5 patients (17.2%), the disease was confined to the terminal ileum; in 17 patients (58.6%), it was extended to the cecum; and in 7 patients (24.1%), there was an ileocolic involvement. Moreover, 18 patients (62.1%) presented stenosis, 6 patients (20.7%) presented stenosis combined with fistula, and the 5 patients (17.3%) that presented inflammatory disease were not responsive to medical treatment. The SES-CD, calculated before surgery, was severe in 12 patients (41.4%), moderate in 15 (51.7%), and mild in only 2 (6.9%). While PCDAI was severe in 13 patients (44.8%), it was moderate in 7 (24.1%) and mild in 9 (31%) (Table 3).

Overall, 18 patients (62.1%) underwent right hemicolectomy and 11 (37.9%) ileocolic resections with a mean of 33 cm of ileum resected (range: 12–50 cm). A laparoscopic approach was used in the majority of cases (65.5%, 19 patients), while the remaining cases (20.6%, 6 patients) were treated with an open approach. The conversion rate was 13.7% (n = 4); one half due to the presence of massive adhesions and the other half due to the presence of abscess (Table 4). The complication rate was 6.9% (n = 2); one patient had a surgical site infection, and the other one had an anastomotic leakage. Anastomosis was performed in each patient without the need for a temporary ostomy. No deaths were recorded.

### 3.1. Endpoints

#### 3.1.1. Primary Endpoint

**Figure 1:**
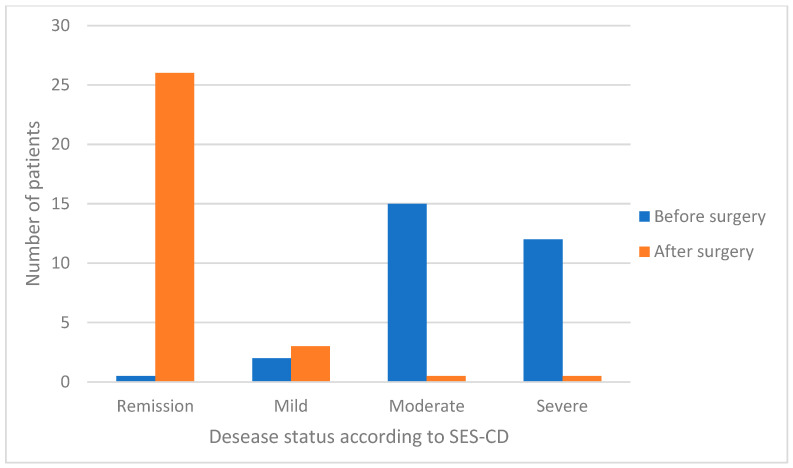
SES-CD before and after surgery.

**Figure 2:**
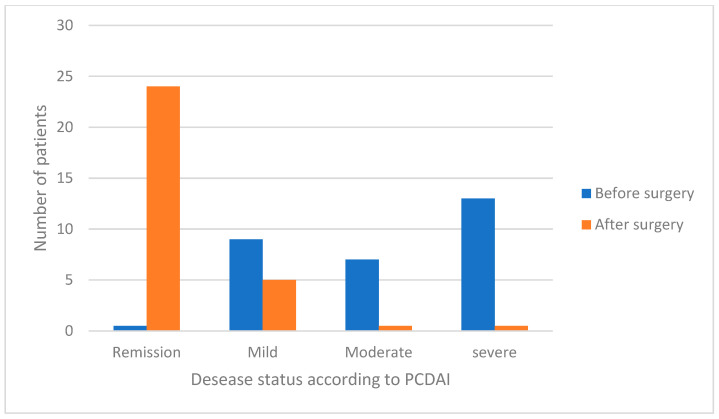
PCDAI before and after surgery.

At the end of follow-up (mean 39.56 months, SD 20.81), SES-CD and PCDAI were calculated for each patient. The endoscopic score (SES-CD) significantly decreased from 12 (median, range 1–15) to 0 (median, range 0–6) (*p* < 0.0001). In particular, it was severe in 0 patients (0%), moderate in 0 (0%), mild in 3 (10.3%); there was remission in 26 (89.7%). The clinical one (PCDAI) decreased from 30 (median, range 10–50) to 0 (median, range 0–15) (*p* < 0.0001). In particular, it was severe in 0 patients (0%), moderate in 0 (0%), mild in 5 (17.2%); there was remission in 24 (82.8%) (Figure 1 and Figure 2).

#### 3.1.2. Secondary Endpoint

**Figure 3:**
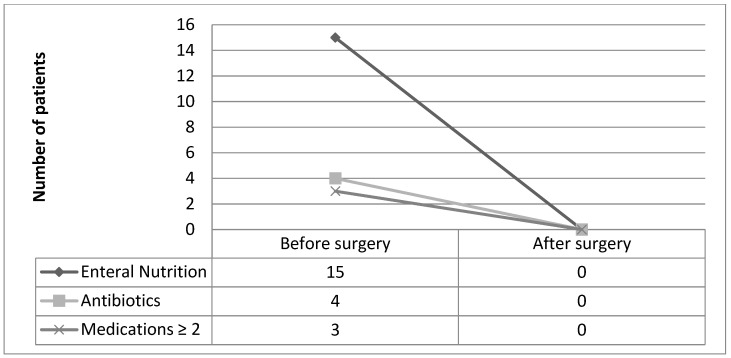
Number of patients under medical therapy before and after surgery.

Moreover, the rate of patients under enteral nutrition decreased from 51.7% (n = 15) before surgery to 0% after surgery (*p* = 0.0001). The rate of antibiotic use decreased from 13.8% (n = 4) to 0% (*p* = 0.0001). The rate of patients under ≥2 medications decreased from 10.3% (n = 3) to 0% (*p* = 0.0001) (Figure 3). The use of anti-inflammatories (corticosteroids or 5-aminosalicylic acid), azathioprine, and biological therapies (infliximab and adalimumab) showed no statistically significant alteration (Table 5). Only one patient (3.45%) showed endoscopic recurrence resistant to medical treatment after surgery.

## 4. Discussion

CD has an important clinical relevance, with a significant incidence and prevalence worldwide [2,21]. Reduced quality of life and high health care costs are the main problems of this disease [22,23]. In our study, we observed a significant improvement in the values obtained by SES-CD and PCDAI before and after surgical treatment. Likewise, three additional parameters examined (the use of enteral nutrition, the use of antibiotics, and the treatment with more than two drugs) showed a clear change after surgical treatment. These data corroborate the hypothesis according to which a surgical treatment can be considered a valid therapeutic option, greatly improving the quality of life of patients.

Up to the present day, surgery has been considered mostly in patients with acutely complicated diseases. One of the cornerstones of therapy in patients with moderate to severe IBD is anti-tumor necrosis factor α [TNF] agents. Nevertheless, approximately 30–40% of patients may not respond to these drugs, and a further 30–40% of patients may lose response over time or may be intolerant to anti-TNFs [24]. In addition to these, other biologic drugs have been approved by the FDA (Food and Drug Administration) for the treatment of IBD. Although there are excellent results, they are not free from side effects and complications, such as common and opportunistic infections, increased risk of lymphoma or melanoma, development of psoriatic, lupus-like reactions, and demyelinating processes. In addition, metabolic and hematological side effects have been found in most patients treated with biological drugs (liver enzyme abnormalities, neutropenia, and lipid profile imbalances) [25]. Also, it is necessary to emphasize the high cost of these therapeutic treatments for the health care system. [26].

In a 2021 review, Kelm et al. agreed on the need to set patients’ quality of life and disease remission as a therapeutic objective, using clinical and endoscopic scores.

Several examined studies confirm that the surgical approach improves quality of life by decreasing the need for immunosuppressive drugs, and it is a safe approach with low rates of postoperative complications and morbidity [27].

The LIR!C trial compared laparoscopic ileocecal resection versus Infliximab for patients with localized non-stricturing CD failing conventional therapy. After 12 months, the results showed that patients undergoing surgery had a similar quality of life as those treated with Infliximab. Moreover, low morbidity rates were reported, showing surgery as a safe procedure [16]. A detailed cost analysis of both procedures was performed by the same group, and it was observed that laparoscopic ileocecal resection was a more cost-effective alternative compared with Infliximab [28]. Finally, the long-term results of the study were examined. After an average follow-up of 63.5 months, no patients in the resection group required a second resection, and only a minority of them who presented disease recurrence needed anti-TNF therapy; while in the Infliximab group, one-half of the patients required an ileocecal resection and the other remained on biologic therapy [29]. A further recent study compared early ICR with anti-TNF therapy, demonstrating that resection was associated with a 33% risk reduction in the rate of the composite outcome of ≥1 of the following: hospitalization, systemic corticosteroid use, CD-related major surgery, and perianal CD [30].

When should surgery be proposed to the patients? Several studies have spoken about “early surgery”. Unfortunately, there is no clear definition of this term, as some studies classify it by considering a time span from diagnosis, while others refer to disease progression that is highly variable. Therefore, we think it might be more appropriate to refer to “early surgery” in patients with: localized luminal disease refractory to medical therapy, stricturing or penetrating phenotype at the time of diagnosis, ASA (American Society of Anesthesiology) Score I or II, good nutritional status and normal weight, no previous or recent use of corticosteroids, and patients without previous large incision from open abdominal surgery [31].

The concept of timing for surgery has been analyzed in several studies. A multicenter study from the Netherlands evaluated the prognosis of patients undergoing primary ICR and demonstrated the lowest incidence of endoscopic recurrence, an escalation of IBD medication, and resection in patients undergoing ICR shortly after diagnosis (0–1 month) [32]. In the literature, another study evaluated two groups of patients with terminal ileitis: one undergoing primary ICR and the other primary medical therapy followed by surgery. After a two-year follow-up, they demonstrated that ICR is safe and effective in patients with localized CD, as patients undergoing primary surgery required significantly fewer anti-inflammatory drugs than patients undergoing surgery in the next stage of the disease [33].

A SURGICROHN—LATAM study conducted in Latin America, compared patients undergoing surgery for early disease (Early Crohn’s Disease—ECD) and those for complications of CD (Complicated Crohn’s Disease—CCD). The latter had worse postoperative outcomes. [34]. Likewise, Avellaneda et al. conducted a second study in Denmark, showing that the CCD group had longer operative times, high conversion rates, and an inferior laparoscopic approach. Furthermore, the CCD group had higher reoperation and rehospitalization rates [35].

Regarding long-term follow-up post-ICR, Dreznik et al. found that clinical recurrence following ICR was present in 11.4% and 28.6% in the first two and five years, and patients treated with early anti-TNF following ICR tend to have less recurrence [36].

Meanwhile, Kotze et al. observed that patients who underwent surgery after >5 years of disease had higher rates of postoperative medical and surgical complications, reoperations, surgical site infections, anastomotic leaks, and abdominal abscesses, compared with patients operated on earlier in the disease course [37].

Many times, physicians have tried to use all available medical treatments before requiring surgery, even in patients with refractory disease. In this regard, biological therapy represents an important therapeutic tool, but if not introduced in a timely manner, it can lead to delays in surgery and more difficult operations, burdening major postoperative complications. The ideal approach should be a personalized therapy for each patient, especially considering the characteristics and phenotype of disease, any risk factors, and personal preferences [31].

The main hurdle to the surgical approach remains the skepticism and the patients’ fear toward surgery. It is very important to set up correct and exhaustive counseling with patients and their families. Furthermore, in order to achieve optimal disease management, a multidisciplinary approach would be necessary, with a close collaboration between gastroenterologists and surgeons.

Our study has some limitations, such as its retrospective nature and small sample size. The group of patients studied was quite homogeneous, being young patients with localized disease, although there is heterogeneity of medical treatment before surgery. Furthermore, it was not easy to characterize surgery as “early” as nowadays, there is no universally accepted definition of the same by the scientific community.

## 5. Conclusions

In our study, we found that surgery in patients with localized CD is a safe and effective approach and can be considered an excellent therapeutic strategy. The two parameters examined, SES-CD and PCDAI, demonstrated a clear improvement in the endoscopic images and disease activity. Moreover, in the postoperative period, we reported a reduction in the use of enteral nutrition, antibiotics, and the combination of more than two drugs. Although surgery is still considered with fear and uncertainty by some patients, in the near future, with the improvement of minimally invasive techniques and a greater awareness of the risks and benefits associated with it, it may represent a valid therapeutic option in patients with localized disease. Finally, as a future line of research, it would be interesting to compare our case series with a group of patients undergoing medical therapy alone.

## Figures and Tables

**Table 1:** Definition of Simple Endoscopic score for Crohn’s Disease.

*Variable*	*0*	*1*	*2*	*3*
***Size of ulcers***	None	Aphthous ulcers (Ø 0.1 to 0.5 cm)	Large ulcers (Ø 0.5 to 2 cm)	Very large ulcers (Ø > 2 cm)
***Ulcerated surface***	None	<10%	10–30%	>30%
***Affected surface***	Unaffected segment	<50%	50–75%	>75%
***Presence of narrowings***	None	Single, can be passed	Multiple, can be passed	Cannot be passed

Ø = Diameter.

**Table 2:** Pediatric Crohn’s Disease Activity Index.

Category	Parameter	Detailed Description	Point
***History (recall, 1 wk)***	Abdominal pain	None	0
		Mild (brief, does not interfere with activities)	5
		Mod/severe (daily, longer lasting, affects activities, nocturnal)	10
	Stools (per day)	0–1 liquid stools, no blood	0
		Up to 2 semi-formed with small blood, or 2–5 liquid	5
		Gross bleeding, or ≥6 liquid, or nocturnal diarrhea	10
***Patient functioning, general wellbeing (recall, 1 wk)***	No limitation of activities		0
	Occasional difficulty in maintaining age-appropriate activities		5
	Frequent limitation of activity, very poor		10
***Laboratory***	Hematocrit (%) (use age-specific reference)	Normal	0
		Mild decrease	2.5
		Mod/severe decrease	5
	Erythrocyte sedimentation rate (mm/h)	<20	0
		20–50	2.5
		>50	5
	Albumin (g/dL)	≥3.5	0
		3.1–3.4	5
		≤3.0	10
***Examination***	Weight	Weight gain or voluntary weight stable/loss	0
		Involuntary weight stable, weight loss 1–9%	5
		Weight loss ≥ 10%	10
	Height at diagnosis	<1 channel decrease	0
		≥1, <2 channel decrease	5
		≥2 channel decrease	10
	Height follow-up	Height velocity ≥ −1 SD	0
		Height velocity < −1 SD, >−2 SD	5
		Height velocity ≤ −2 SD	10
	Abdomen	No tenderness, no mass	0
		Tenderness, or mass without tenderness	5
		Tenderness, involuntary guarding, definite mass	10
	Perirectal disease	None, asymptomatic tags	0
		1–2 Indolent fistula, scant drainage, no tenderness	5
		Active fistula, drainage, tenderness, or abscess	10
	Extraintestinal manifestations (n)	0	0
		1	5
		≥2	10

**Table 3:** General features of study patients.

Parameter	Category	Value
Sex, n (%)	Male	19 (65.5)
	Female	10 (34.5)
Age at diagnosis, years	**Median (range)**	**12 (5–16)**
Age at surgery, years	**Median (range)**	**14 (8–20)**
Site of disease, n (%)	Ileal	5 (17.2)
	Ileocecal	17 (58.6)
	Ileocolic	7 (24.1)
Indication for surgery, n (%)	Stenosis	18 (62.1)
	Fistula	0
	Stenosis and fistula	6 (20.7)
	Unresponsive to medical treatment	5 (17.3)
Medical treatment, months	**Median (range)**	**10 (1–144)**
SES-CD ^1^	**Median (range)**	**12 (1–15)**
	Mild, n (%)	2 (6.9)
	Moderate, n (%)	15 (51.7)
	Severe, n (%)	12 (41.4)
PCDAI ^2^	**Median (range)**	**30 (10–50)**
	Mild, n (%)	9 (31.0)
	Moderate, n (%)	7 (24.1)
	Severe, n (%)	13 (44.8)

^1^ Simple Endoscopic Score for Crohn’s Disease; ^2^ Pediatric Crohn’s Disease Activity Index.

**Table 4:** Intraoperative and perioperative details of the study population.

Parameter	Category	Value
Surgical technique (n,%)	Right emicolectomy	18 (62.1)
	Ileocolic resection	11 (37.9)
Surgical approach (n,%)	Open	6 (20.6)
	Videolaparoscopy	19 (65.5)
	Conversion	4 (13.7)
Complication rate (n,%)		2 (6.9)

**Table 5:** Results of surgery after follow-up ≥6 months.

Parameter	Category	Before Surgery	After Surgery	*p* Value
SES-CD ^1^	**Median (range)**	**12 (1–15)**	**0 (0–6)**	**<0.0001**
	Remission (<3), (n, %)	0	26 (89.7)	**0.0001**
	Mild (3–6), (n %)	2 (6.9)	3 (10.3)	0.639
	Moderate (7–14), (n. %)	15 (51.7)	0	**0.0001**
	Severe (≥15), (n. %)	12 (41.4)	0	**0.0001**
p-CDAI ^2^	**Median (range)**	**30 (10–50)**	**0 (0–15)**	**<0.0001**
	Remission (<10), (n. %)	0	24 (82.8)	**0.0001**
	Mild (10–27), (n. %)	9 (31.0)	5 (17.2)	0.219
	Moderate (28–37), (n. %)	7 (24.1)	0	**0.0048**
	Severe (≥38), (n. %)	13 (44.8)	0	**0.0001**
Medical Therapy	Enteral nutrition (n. %)	15 (51.7)	0	**0.0001**
	Antiobiotics (n. %)	4 (13.8)	0	**0.0382**
	Antinflammatory (n. %)	2 (6.9)	6 (20.7)	0.128
	Azathioprine (n. %)	6 (20.7)	6 (20.7)	1.0
	Biological therapies (n. %)	19 (65.5)	17 (58.6)	0.588
	Medications ≥2 (n. %)	3 (10.3)	0	**0.0001**

^1^. Simple Endoscopic Score for Crohn’s Disease; ^2^. Pediatric Crohn’s Disease Activity Index.

## Data Availability

The original contributions presented in the study are included in the article, further inquiries can be directed to the corresponding author.
